# 2D-DIGE proteomic analysis of *vastus lateralis* from COPD patients with low and normal fat free mass index and healthy controls

**DOI:** 10.1186/s12931-017-0525-x

**Published:** 2017-05-03

**Authors:** Ramzi Lakhdar, Ellen M. Drost, William MacNee, Ricardo Bastos, Roberto A. Rabinovich

**Affiliations:** 1 ELEGI Colt Laboratory, Centre for Inflammation Research, The Queen’s Medical Research Institute, University of Edinburgh, 47 Little France Crescent, Edinburgh, EH16 4TJ Scotland, UK; 2 Institut d’Investigacions Biomèdiques August Pi i Sunyer (IDIBAPS), University of Barcelona, Barcelona, Spain

**Keywords:** COPD, Skeletal muscle dysfunction/wasting, Proteomic analysis, 2D-DIGE, DOT1L, Ageing

## Abstract

**Background:**

Chronic obstructive pulmonary disease (COPD) is associated with several extra-pulmonary effects of which skeletal muscle wasting is one of the most common and contributes to reduced quality of life, increased morbidity and mortality. The molecular mechanisms leading to muscle wasting are not fully understood. Proteomic analysis of human skeletal muscle is a useful approach for gaining insight into the molecular basis for normal and pathophysiological conditions.

**Methods:**

To identify proteins involved in the process of muscle wasting in COPD, we searched differentially expressed proteins in the *vastus lateralis* of COPD patients with low fat free mass index (FFMI), as a surrogate of muscle mass (COPD_L,_ *n* = 10) (FEV_1_ 33 ± 4.3% predicted, FFMI 15 ± 0.2 Kg.m^−2^), in comparison to patients with COPD and normal FFMI (COPD_N,_ *n* = 8) and a group of age, smoking history, and sex matched healthy controls (C, *n* = 9) using two-dimensional fluorescence difference in gel electrophoresis (2D-DIGE) technology, combined with mass spectrometry (MS). The effect of silencing DOT1L protein expression on markers of cell arrest was analyzed in skeletal muscle satellite cells (HSkMSCs) in vitro and assessed by qPCR and Western blotting.

**Results:**

A subset of 7 proteins was differentially expressed in COPD_L_ compared to both COPD_N_ and C. We found an increased expression of proteins associated with muscle homeostasis and protection against oxidative stress, and a decreased expression of structural muscle proteins and proteins involved in myofibrillogenesis, cell proliferation, cell cycle arrest and energy production. Among these was a decreased expression of the histone methyltransferase DOT1L. In addition, silencing of the DOT1L gene in human skeletal muscle satellite cells in vitro was significantly related to up regulation of p21 ^WAF1/Cip1^/CDKN1A, a marker of cell arrest and ageing.

**Conclusions:**

2D-DIGE coupled with MS identified differences in the expression of several proteins in the wasted *vastus lateralis* that are relevant to the disease process. Down regulation of DOT1L in the *vastus lateralis* of COPD_L_ patients may mediate the muscle wasting process through up regulation of markers of cell arrest and senescence.

**Electronic supplementary material:**

The online version of this article (doi:10.1186/s12931-017-0525-x) contains supplementary material, which is available to authorized users.

## Background

Chronic obstructive pulmonary disease (COPD) is characterized by airflow limitation that is not fully reversible [1], is usually progressive and associated with a chronic inflammatory response of the lungs to noxious particles or gases of which cigarette smoking is the most common risk factor [2].

COPD has significant systemic effects, among which muscle wasting is common, and has been extensively studied [3, 4]. Muscle wasting results in a loss of muscle strength [3, 5–8], contributes to reduced exercise capacity [9–12] and is a predictor of health related quality of life (HRQoL) [13] and survival [14, 15] independent of the degree of airflow limitation [12].

The mechanisms underlying weight loss and muscle wasting in COPD are not completely understood and are likely to be multi-factorial, including low physical activity in patients with a sedentary habit, oxidative stress and inflammation, among others [16, 17]. Results of a previous study of a microarray analysis of the *vastus lateralis* of COPD patients with muscle wasting, showed over expression of the cyclin-dependent kinase inhibitor 1A (p21 ^WAF1/Cip1^/CDKN1A) and changes in expression of genes associated with cell cycle arrest, growth regulation and energy production [18]. These results suggest that cell senescence may play a role in muscle atrophy in COPD [18].

There is evidence that COPD is a disease of accelerated ageing [19]. Animal models of premature ageing show structural changes in the lung that resemble those in COPD and also show skeletal muscle abnormalities [20] that occur with ageing [21]. It has been reported that limb muscles of patients with COPD have increased number of senescent satellite cells and a decreased muscle regenerative capacity, compromising the maintenance of muscle mass in these individuals [22]. Thus premature cellular senescence and subsequent exhaustion of the muscles regenerative potential may be related to the muscle abnormalities that are characteristic of these patients.

Proteomic analysis is a powerful tool for global evaluation of protein expression, and a useful approach, coupled with other functional genomic approaches, to gain insight into the molecular basis for normal and pathophysiological conditions [23, 24]. The two-dimensional fluorescence difference in gel electrophoresis (2D-DIGE) technology is now recognized as an accurate method to determine and quantify proteins [25, 26], and therefore to assess changes in protein expression associated with disease phenotypic states.

With the aim of identifying proteins that are potentially involved in the process of muscle wasting in COPD, we searched for differentially expressed proteins in the *vastus lateralis* of COPD patients with low fat free mass index (FFMI), as a surrogate of muscle mass, (COPD_L_) in comparison to patients with COPD and normal FFMI (COPD_N_) and a group of age and sex matched healthy controls (C) using 2D-DIGE combined with mass spectrometry (MS).

Among the proteins identified, the histone methyltransferase DOT1L was found to be down-regulated in COPD_L_. We hypothesize that down-regulation of DOT1L mediates cell senescence through the up regulation of molecules involved in cell arrest (i.ep21 ^WAF1/Cip1^/CDKN1A). To test this hypothesis, DOT1L gene was silenced in vitro in Human Skeletal Muscle Satellite Cells and mRNA and protein levels of p21 ^WAF1/Cip1^/CDKN1A was assessed.

## Methods

### Study Group

Eighteen stable patients with COPD, ten with low FFMI (COPD_L_) and eight with normal FFMI (COPD_N_), and nine age, gender and smoking status-matched healthy subjects with normal FFMI were included in the present study (Table 1). The COPD patients had a history compatible with the disease: at least 10 pack/years of smoking and evidence of chronic airflow limitation (post bronchodilator FEV_1_/FVC < 0.7) [27]. All participants were informed of any risks and discomfort associated with the study, and written informed consent was obtained. The study was approved by the Lothian Regional Ethics Committee.

**Table 1:** Characteristics of the study groups

	Controls		COPD_N_		COPD_L_		*p*-value
M/F	8/1	A	6/2	A	8/2	A	ns
Age (Years)	68.8 ± 4.4	A	68.6 ± 5.2	A	66.7 ± 5.9	A	ns
Height (m)	1.74 ± 0.08	A	1.70 ± 0.09	A	1.67 ± 0.08	A	ns
Weight (Kg)	89.93 ± 15.63	A	76.1 ± 12.65	A	51.71 ± 5.71	B	<0.001
BMI (Kg.m^−2^)	29.3 ± 4.6	A	26.2 ± 2.4	A	18.9 ± 1.9	B	<0.0001
FFM	62.55 ± 11.35	A	54.13 ± 10.13	AB	43 ± 5.69	C	<0.001
FFMI (Kg.m^−2^)	20.5 ± 2.3	A	18.6 ± 1.6	B	15.3 ± 0.7	C	<0.0001
Active/ex-smokers	1/8	A	2/6	A	2/8	A	ns
Pack/Year	30.9 ± 15	A	50.9 ± 23.1	A	64.3 ± 39.8	A	ns
Average cessation (years)	24.6 ± 16.8	A	7.9 ± 7.6	B	5.4 ± 7.4	B	<0.05
Age at smoking cessation (years)	44.2 ± 15.3	A	60.8 ± 8.3	B	61.3 ± 7.8	B	0.0039
mMRC			2.1 ± 1.4		3.1 ± 1.1		ns
FEV_1_ (L)	2.9 ± 0.5	A	1.2 ± 0.5	B	0.9 ± 0.4	B	<0.0001
FEV_1_ (% pred)	98.6 ± 12.1	A	44.3 ± 19.9	B	33.2 ± 13.7	B	<0.0001
FVC (L)	4 ± 0.7	A	2.9 ± 1.1	B	2.6 ± 1	B	<0.01
FVC (% pred)	105.8 ± 12.4	A	85.6 ± 31.5	AB	76.5 ± 18.2	B	<0.05
FEV_1_/FVC	0.7 ± 0	A	0.4 ± 0.1	B	0.3 ± 0.1	B	<0.0001
PaO_2_ (mmHg)	74.3 ± 8	A	68.5 ± 9.7	A	75.2 ± 13.1	A	ns
PaCO_2_ (mmHg)	41.2 ± 2.4	A	41 ± 3.9	A	43.1 ± 5.2	A	ns
Physical Activity (V)	11.9 ± 5.1	A	6.3 ± 6	AB	1.1 ± 1	B	<0.0005
Physical Activity (L)			33.1 ± 16	A	43.5 ± 10.4	A	ns
QMVC (N)	372.3 ± 89	A	317.9 ± 89.9	A	202.2 ± 51.8	B	<0.005
6MWD (m)	569.3 ± 62.4	A	390 ± 170.2	B	327 ± 134.1	B	<0.005
Exacerbation			1.8 ± 1.5	A	4 ± 2.2	B	<0.05
BODE			4.3 ± 3	A	6.2 ± 2.3	A	ns
SGRQ Symptoms			62.9 ± 10.1	A	78.3 ± 15.2	B	<0.05
SGRQ Activity			56.9 ± 31	A	86.2 ± 13.4	B	<0.05
SGRQ Impact			37.8 ± 25	A	59.5 ± 20.8	A	ns
SGRQ Total			47.9 ± 21.8	A	70.7 ± 16.4	B	<0.005
Type I Fibre (%)	38.5 ± 11	A	26 ± 9.6	A	24.7 ± 13.5	A	ns
Type II area (μ2)	2564 ± 783.8	AB	3096 ± 893.6	A	2034 ± 498.8	B	<0.05

*Abbreviations*: *COPD*_*N*_ COPD patients with normal, *FFMI* COPD_L_ patients with COPD with low FFMI, *BMI* Body mass index, *FFM* fat free mass, *FFMI* fat free mass index, *MRC* medical research council dyspnoea score, *FEV*_*1*_ forced expiratory volume in the first second, *FVC* forced vital capacity, *PaO*_*2*_ arterial oxygen partial pressure, *PaCO*_*2*_ arterial carbon dioxide partial pressure, *Physical Activity (V)* Voorrips Questionnaire, *Physical activity (L)* London Chest Activity of Daily Living Scale, *QMVC* quadriceps maximal voluntary contraction, *6MWD* six minute walking distance, *SGRQ* St. George’s Respiratory Questionnaire, *ns* not significant, *NA* not applicable. Comparisons among groups were done using ANOVA and Student-Newman-Keuls as a post-hoc test. Differences among the three different groups were stated using letters A,B and C where sharing a letter implies no differences between these groups and having a different letter implies a statistical difference in the post-hoc test

## Measurements

Spirometry was measured (Alpha Spirometer; Vitalograph, Buckingham, UK) according to American Thoracic Society/European Respiratory Society (ATS/ERS) standards in all subjects [28] before and after the administration of 2.5 mg of nebulised salbutamol. Arterial blood gases were measured (Ciba Corning 800, USA). Body composition was estimated by a bioelectric impedance device (TBF-300 M, TANITA Corporation, Tokyo, Japan). Low FFMI was defined as <16 kg.m^−2^ for male and < 15 kg.m^−2^ for female COPD patients [29]. As a measure of exercise tolerance all participants performed an encouraged 6MWT according to ATS guidelines [30]. Muscle strength was assessed as the maximal isometric quadriceps voluntary contraction (QMVC) using a strain gauge dynamometer (Chatillon® K-MSC 500, Ametek, Florida) [31]. HRQoL was assessed using the St. George’s Respiratory Questionnaire [32]. Physical activity level was assessed using the Voorrips physical activity questionnaire in the whole population participating in the study [33]. In COPD patients activities of daily living were assessed by the London Chest Activity of Daily Living Scale (LCADL) [34].

### Muscle biopsy

An open muscle biopsy of the *vastus lateralis* (~0.8 g) was taken in a Clinical Research Facility bed area using a standard surgical technique [18].

### Fibre type typification

Paraffin sections (5um) were de-waxed and re-hydrated through graded ethanol using standard procedures. Sections were placed in 250 ml of Novocastra pH8 retrieval buffer and subjected to antigen retrieval in a de-cloaking chamber (Biocare Medical, USA) using a protocol described elsewhere [35].

#### *Vastus lateralis* muscle protein extraction

*Vastus lateralis* muscle (~0.1 g) from each sample was cut into small pieces with a scalpel and transferred in metal bead tubes. 50 μl of extracting buffer (0.3% Sodium carbonate, 0.5% Sodium hydrogen carbonate and 0.6% CHAPS, containing phosphatase inhibitors, protease inhibitors and benzonase) was added to 15 mg of each tissue sample. Protein concentration was determined by the Bradford method (Bio-Rad Laboratories, Hercules, CA).

### Two-dimensional difference gel electrophoresis (2D-DIGE)

**Fig. 1:**
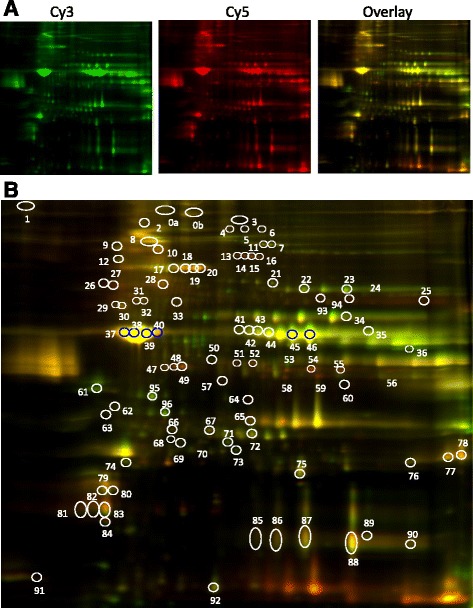
*Vastus lateralis* protein expression profiling by 2D DIGE. Representative 2D-DIGE images (from Gel1) showing differentially expressed protein spots. **a** 2D images of two samples; COPD_L_ and COPD_N_ subjects; respectively labelled with Cy3 (*green spots*, COPD_N_) and Cy5 (*red spots*, COPD_L_) and the corresponding overlap, generated by ImageQuant software (pH range 4–9 from left to right in the horizontal dimension; MW range 15 kDa-150 kDa from bottom to top in the vertical dimension). **b** Images were further analyzed by DeCyder Image analysis software to detect the differentially expressed protein spots (*white circles*). *Purple circles* correspond to spots not include in the final selection

2D-DIGE analysis including protein labeling, 2D-electrophoresis, gel analysis and identification of proteins of interest by mass spectrometry were performed by Applied Biomics (Hayward, CA) using established protocols (Fig. 1).

### Cell culture

Human Skeletal Muscle Satellite Cells (HSkMSCs) were purchased from Innoprot (SKELETAL MUSCLE INNOPROFILE™, REF: P10976). These cells were isolated by ScienCell Research Laboratories from human muscle of the pectoral girdle. Cell culture media kits were purchased from the same company (Innoprot REF: P60132). The satellite cells were grown to confluent myoblasts in poly-L-lysine coated flasks (2 μg/cm2, T-75 flask) according to the manufacturer’s guide. Myoblasts were differentiated into myotubes as previously described [36].

### DOT1L gene kockdown in HSkMSC by siRNA

#### siRNA design

For siRNA-mediated down-regulation of DOT1L, a specific validated target sequence was purchased from Ambicon, Life Technologies (Silencer® siRNA (DOT1L, siRNA ID: 112262) and transient transfection of siRNA was performed. siRNA experiments were conducted using a stock solution of 10uM siRNA at a final concentration of 30 pmol duplex siRNA per well in a six well plate following the manufacture protocol. For transfection into the cells, Lipofectamine® 2000 Transfection Reagent (Ambicon, Life Technologies) was used according to the manufacturer’s protocol.

#### siRNA cell transfection

Human skeletal muscle satellite cells were transferred in six well plates at 2–3 10^4^ cells/cm^2^. At 70–80% confluency, myoblasts were transfected following the manufacturer’s guide and as previously described [37, 38].

### Quantitative RT-PCR

Total cellular RNA was prepared from the cells using an NucleoSpin® RNA kit (Macherey-Nagel, Fisher Scientific UK) and 0.5 ug of the RNA was then reverse transcribed to cDNA using a High Capacity cDNA Reverse Transcription Kit (Applied Biosystems). For quantitative RT-PCR analysis, the cDNA was combined with gene-specific forward and reverse primers for DOT1L and p21 ^WAF1/Cip1^/CDKN1A a SYBR Green PCR master mix and subjected to real time fluorescence detection PCR using an ABI Prism 7900 Sequence Detection System (Applied Biosystems, Foster City, CA, USA).

### Western Blot Analysis

Cells were harvested and protein concentration was determined. DOT1Landp21 ^WAF1/Cip1^ protein level was determined by immunoblotting using antibodies against DOT1L (Novus Biologicals, NB100-40845) and p21 ^WAF1/Cip1^ (ab79601) (Abcam, Bristol, UK).

### Statistical analysis

For 2D-DIGE proteomic analysis, the data were analysed using Student-*t* test to compare between the patient groups (COPD_N_, COPD_L_) and the healthy controls. Gene expression q-PCR data and immunoblotting results for DOT1Landp21 ^WAF1/Cip1^/CDKN1A after gene knockdown are expressed as mean ± SEM. Comparisons between the groups were performed using Mann–Whitney U test for non-parametric variables. Correlation analysis between variables was conducted using Pearson’s correlation index for continuous variables. A *p* value <0.05 was taken as statistically significant. The statistics were conducted using the statistical package SAS version 9.3 (SAS Institute Inc, Cary, NC, USA).

## Results

The anthropometric characteristics and pulmonary function data of study subjects are depicted in Table 1. Both groups of COPD patients had chronic airflow limitation compared to healthy controls (C) all of who had normal spirometry, but there were no differences in spirometry between COPD_N_ and COPD_L_.

Compared to COPD_N_, COPD_L_ had significantly lower BMI, FFM and FFMI (as expected by the study design), poorer HRQoL with higher values in all of the domains of the St. George’s respiratory questionnaire, and worse muscle function as assessed by QMVC. No statistical differences in physical activity measured by the Voorrips questionnaire (PAV) or activities of daily living (ADL) assessed with the LCADL (PAL) were seen between the COPD groups although a trend towards lower PAL was observed in COPD_L_.

Both COPD groups showed a redistribution of muscle fibre type with a higher proportion of type II fibres and a lower proportion of type I in comparison to healthy controls; however this did not reach statistical significance. Type II fibre area was significantly reduced in COPD_L_ in comparison with COPD_N_ (Table 1).

### Proteomic analysis

To search for differentially expressed proteins (DEPs), we performed three pair-wise class comparisons: COPD_L_ vs. COPD_N_, COPD_L_ vs. C and COPD_N_ vs. C. A list with 96 protein spots was selected from the analysis using the DeCyder software. These spots corresponded to the most prominent changes in terms of fold-change and/or statistical significance that could be detected. Among this list, there were some significant changes in protein expression in the paired groups (*p* < 0.05): 50 spots were differentially expressed comparing COPD_N_ and Controls, whereas, 41 spots were found differentially expressed comparing COPD_L_ and the Control group and 37 spots when comparing COPD_L_ and COPD_N_ (Additional file 1: Table S1).

In order to select the most relevant DEPs related to muscle wasting in COPD, we focused on the list of 37 DEPs between COPD_L_ and COPD_N_. To strengthen the biological relevance of these proteins, we further selected from this list 20 spots that were also differentially expressed between COPD_L_ and C. We further excluded proteins that were differentially expressed between COPD_N_ and the control group, as both subgroups have normal muscle bulk and these proteins may not be relevant to the process of muscle wasting. The number of spots was therefore reduced to 11.

These spots were further extracted from the gel and the proteins identified by mass spectrometry (Table 2).

**Table 2:** Proteins differentially expressed between COPD_L_vs COPD_N_and COPD_L_vs C groups

				COPD_L_vs COPD_N_		COPD_L_vs C		
Assigned spot number		Protein name	Protein symbol	*P* value	Av. Ratio	*P* value	Av. Ratio	Protein relevant function
17		Serum albumin	ALBU	**0.0053**	1.67	**0.022**	1.36	Transportation of substances in the blood.
67	Up regulated	Heat shock protein beta-1	HSPB1	**0.011**	1.3	**6.7E-05**	1.48	Heat shock, ROS scavenger
75		Alpha-crystallin B chain	CRYAB	**0.017**	1.38	**0.00026**	1.53	Heat shock, muscle homeostasis
1		Histone-lysine N-methyltransferase, H3 lysine-79 specific	DOT1L	**0.0065**	−2.25	**0.014**	−1.45	DNA repair, deficiency leads to cell arrest
91	Down regulated	Myosin light chain 1/3, skeletal muscle isoform	MYL1	**0.049**	−2.13	**0.0048**	−2.38	Myosin light chain expressed in Type II fibres, muscle cell proliferation
48		Troponin T, slow skeletal muscle	TNNT1	**0.025**	−2.6	**0.023**	−2.21	Component of the thin filament of the sarcomere
60		Myozenin-1 OS = Homo sapiens	MYOZ1	**0.00086**	−2.44	**0.029**	−1.63	Promote type II fibres, calcineurin-interacting proteins

Seven proteins were identified that full fill the criteria set for the analysis. Student *t* test was used to compare between different groups, *p*-value <0.05 significant (bold). Av. Ratio: Average. Ratio; fold change a positive value means increased ratio, a negative value means decreased ratio

These spots represented 7 proteins (2 proteins were represented by three different spots). Among these, 3 proteins were up regulated; Serum albumin (ALBU), Heat shock protein beta-1 (HSPB1) and Alpha-crystallin B chain (CRYAB) and 4 proteins Histone-lysine N-methyltransferase, H3 lysine-79 specific (DOT1L), Troponin T, slow skeletal muscle (TNNT1), Myozenin-1 (MYOZ1) and Myosin light chain 1/3, skeletal muscle isoform (MYL1) were down regulated (Table 2).

The up-regulated proteins are involved in protection against oxidative stress (HSPB1) and in muscle homeostasis (CRYAB). Whereas, the down regulated protein are involved in cell cycle regulation (DOT1L), muscle cell proliferation (MYL1), type II fibres promotion (MYOZ1), and are components of the thin filament (TNNT1). DOT1L was down regulated with a significant fold change (FC) in COPD_L_ compared with both COPD_N_ (*P* value = 0.0065, FC = −2.25) and C (*P* value = 0.014, FC = −1.45).

### DOT1L gene knockdown

In our previous microarray analysis we found a differential expression of genes related to premature ageing/cell cycle arrest [18]. Because down regulation of DOT1L has been reported in relation to cell cycle arrest, we explored the effect of silencing DOT1L on cell arrest markers in Human Skeletal Muscle Satellite Cells in vitro, in particular on p21 ^WAF1/Cip1^/CDKN1A that was found up-regulated in our previous study.

**Fig. 2:**
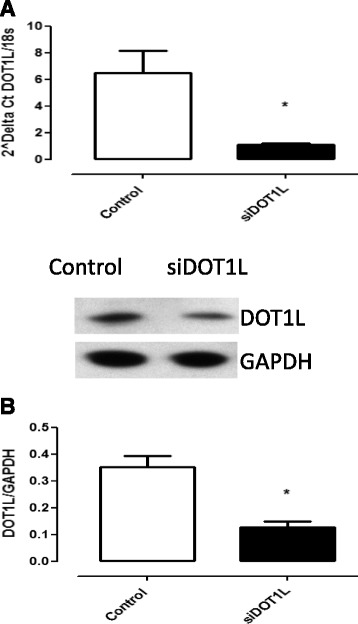
siRNA mediated gene silencing of DOT1L validated by Q-PCR and western blot. The results show a down regulation of DOT1L mRNA expression (**a**) and a decrease of DOT1L protein level (**b**). Empty bars are untreated cells and solid bars are transfected cells. Results from HSkMSC cultures derived from three different experiments on cells at passage three, Graph is presenting means ± SEM. *, *p*-value < 0.05 siRNA treated cells compared to controls

**Fig. 3:**
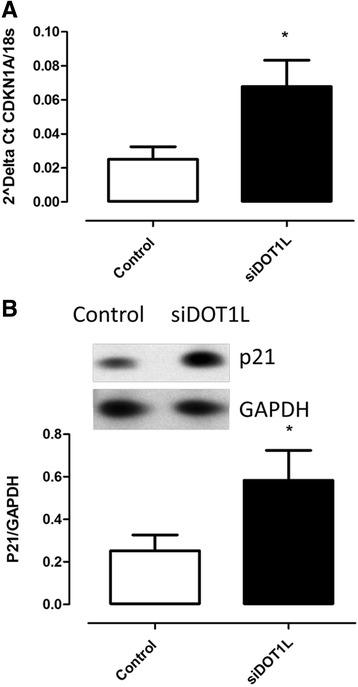
siRNA knockdown of DOT1L up regulates the expression of CDKN1A mRNA (**a**) and p21 protein level (**b**). Empty bars are untreated cells and solid bars are transfected cells. HSkMSC cultures derived from three different experiments on cells at passage three, Graph is presenting means ± SEM. *, *p*-value < 0.05 siRNA treated cells compared to controls

The results confirmed that the siRNA sequence led to significant reduction in DOT1L gene expression, and protein level (Fig. 2) and that the siRNA DOT1L reduced mRNA content was related to an up-regulation of p21 ^WAF1/Cip1^/CDKN1A gene expression and protein content (*P* < 0.05) (Fig. 3).

## Discussion

This study shows changes in protein expression in *vastus lateralis* muscle of COPD patients with skeletal muscle wasting using the highly sensitive 2D-DIGE technique combined with mass spectrometry analysis. We identified up-regulated proteins associated with protection against oxidative stress and muscle homeostasis, and down-regulated proteins involved in cell cycle arrest, growth regulation, energy production and muscle formation. Among the down-regulated proteins was the histone methyltransferase DOT1L, a critical regulator of the cell cycle. In addition, we showed that DOT1L gene silencing in human skeletal muscle satellite cells in vitro is significantly related to up regulation of p21 ^WAF1/Cip1^/CDKN1A, a marker of cell arrest and senescence.

In this analysis, we selected a group of DEPs using restricted criteria to strengthen the biological relevance of these proteins in the process of muscle wasting based on: a) that they were differentially expressed between COPD_L_ and COPD_N_, b) were also differentially expressed between COPD_L_ and C, c) they were not differentially expressed between COPD_N_ and C (both groups with normal FFMI) [18]. These criteria reduced the list of DEPs to a set of seven proteins identified by mass spectrometry potentially involved in the process of muscle wasting in COPD_L_.

One of the proteins whose expression was found significantly decreased in COPD_L_ is DOT1L (disruptor of telomeric silencing-1), an evolutionarily conserved histone methyltransferase that methylates lysine 79 located within the globular domain of histone H3. Methylation of H3K79 is involved in the regulation of telomeric silencing, cellular development, cell-cycle checkpoint, DNA repair, and regulation of transcription [39, 40]. Several studies show that DOT1L is a critical regulator of the cell cycle [41, 42]. Overall, DOT1L is required to maintain genomic and chromosomal stability. It has been shown that deficiency of DOTL1 leads to chromosomal miss-aggregation [43] and that this chromosomal instability leads to cell cycle arrest at the G1 phase and induced senescence, thus disturbing proliferation of human cancer cells [43]. Furthermore, the transcription profiles of DOT1L-deficient mouse embryonic stem cells (ESCs) and their differentiated derivatives contain a high proportion of miss-regulated genes with known functions in cell cycle and cellular proliferation that may represent direct targets of DOT1L regulation [44].

We have previously shown, by different methodologies, an up-regulation of the cyclin-dependent kinase inhibitor p21 ^WAF1/Cip1^ in COPD patients with muscle wasting [18]. p21 ^WAF1/Cip1^protein, encoded by the gene CDKN1A, binds to and inhibits the activity of several cyclin-dependent kinases (CDKs), and thus functions as a regulator of cell cycle progression [45]. In addition to growth arrest, p21 ^WAF1/Cip1^ can mediate cellular senescence [46, 47].

To test the potential association between down-regulation of DOT1L and up-regulation of p21 ^WAF1/Cip1^we used siRNA to silence DOT1L in human skeletal muscle satellite cells (HSkMSCs) and measured the effect on gene expression and protein levels of CDKN1A. Knock down of DOT1L in this cell line resulted in an up-regulation of CDKN1A gene expression and protein levels of p21 ^WAF1/Cip1^.

Our findings are in agreement with previous studies showing an association between DOT1L deficiency and inhibition of cell proliferation due to G0/G1 cell-cycle arrest in MLL-AF9 [48]. A study investigating several cyclin-dependent kinase inhibitors (CKI), (INK4 (p16^INK4a^, p15^INK4b^, p18^INK4c^, and p19^INK4d^) and CIP/KIP (p21^CIP/WAF1^, p27^KIP1^, and p57^KIP2^)) in DOT1L deficient NCI-H1299 cells and A549 cells have shown that DOT1L deficiency up-regulated p21 ^WAF1/Cip1^ expression, but down-regulated other CKIs [43]. The authors suggested that the transcriptional up-regulation of p21 ^WAF1/Cip1^ in DOT1L-deficient A549 cells induced the hypo-phosphorylation of CDK2 and Rb, which inhibits the progression from G1 to S phase [43]. The mechanism by which the DOT1L down regulation interferes with the expression of specific CKIs remains to be elucidated. Also further investigation is needed to shed light on the mechanisms by which DOT1L-down regulation interacts with other factors associated with cell proliferation and cell cycle arrest and regulates senescence in COPD patients with muscle wasting.

In this study, we found an increase of the protein levels of HSPB1 and CRYAB in COPD_L_. HSPB1 participates in the regulation of apoptosis, protects the cell against oxidative stress, and is involved in the regulation of the cytoskeleton [49]. In turn HSPB1 has been related to better fatigue resistance [50] and has been shown to be up-regulated in animal models of diabetes-related muscle weakness [51]. Moreover, it has been reported that the chaperone effect of CRYAB on the cytoskeleton in relation to tubulin/microtubule, is a key mechanism for muscle adaptation, muscle differentiation and protection from atrophy [52]. Increased levels of molecular heat shock proteins reported in this study is in line with well documented evidence of oxidative and nitrosative stress in COPD patient’s peripheral muscle [53–57], especially in patients with low body mass index (BMI) [57] and hypoxemia [53] and may reflect an attempt of the cell to defend itself against these insults.

Our results also revealed an up-regulation of ALBU and down-regulation of other structural proteins MYL1, TNNT1, and MYOZ1 in both COPD_L_ compared to COPD_N_ and COPD_L_ compared to C. The latter is consistent with the fibre type distribution in our population, showing a tendency towards a decrease in Type I fibres, with an increase in the proportion of Type II fibres in the muscle in COPD_L_. The differential expression of structural proteins MYL1, TNNT1, MYOZ1 in our population further highlights the documented structural changes [58] in *vastus lateralis* muscle of COPD patients with muscle atrophy.

### Limitation of the study

In spite of the efforts made in matching the populations, healthy control subjects present differences in fat free mass index (FFMI) compared to the COPD patients with normal FFMI. However, both groups (COPD_N_ and C) have FFMI levels above what is considered normal in these populations. No differences were seen in fat free mass (FFM) or BMI between these groups. As these groups were used to select biological relevant proteins related to muscle wasting from the DEPs between COPD_N_ and COPD_L,_ by excluding from this list those that were not differentially expressed between COPD_N_ and C, we do not think that this affects the conclusions of the present study.

It is worthwhile mentioning that p21 was not identified as an up-regulated protein by the technology used in the present study. We cannot exclude that this protein is among the other differentially expressed proteins detected but still not identified by MS. Although unlikely, another possible explanation is that this protein is not included among the resolved spots in the 2D gels since p21 has a low molecular mass (18 kDa) and the theoretical MW range is 15 kDa-150 kDa from bottom to top. In more general terms, although the 2D-DIGE is a sensitive, accurate and reproducible technique, it faces the inherent limitations of gel-based proteomics, regarding the proteomic coverage. In any event, results previously published by our group have demonstrated, by different methodologies, an up-regulation of p21 both at the gene and protein level in COPD patients with muscle wasting [18].

## Conclusions

Our study showed that 2D-DIGE coupled with MS is useful technique to identify differentially expressed proteins related to muscle wasting in patients with COPD. These results complement our previous findings on the transcriptome of these patients and strengthen the evidence that premature ageing, along with oxidative stress, may play a role in muscle wasting in COPD. p21 ^WAF1/Cip1^/CDKNA1 might represent a target of DOT1L in *vastus lateralis* muscle of COPD patients.

## Additional file

Additional file 1: A detailed methodology is provided: Table S1: Differentially expressed spots between different groups comparing COPDN vs C, COPDL vs C and COPDL vs COPDN using the twodimensional fluorescence difference in gel electrophoresis (2D-DIGE) technology. (DOCX 64 kb)

## Abbreviations

2D-DIGE: Two-dimensional fluorescence difference in gel electrophoresis
6MWD: Six minute walking distance
ADL: Activities of daily living
ANKRD1: Ankyrin repeat domain 1 (cardiac muscle)
ATF3: Activating transcription factor 3
BIA: Bioimpedance analysis
C: Control subjects
CDKN1A: Cyclin-dependent kinase inhibitor 1A (p21 ^WAF1/Cip1^)
CEBPA: CCAAT/Enhancer binding protein (C/EBP), alpha
CKIs: Cyclin-dependent kinase inhibitors
COPD: Chronic obstructive pulmonary disease
COPDL: Patients with COPD and low FFMI
COPDN: Patients with COPD and normal FFMI
DCM: Idiopathic dilated cardiomyopathy
DEPs: Differentially expressed proteins
DNA: Deoxyribonucleic acid
ESCs: Embryonic stem cells
FC: Fold change
FEV_1_: Forced expiratory volume in the first second
FFMI: Fat free mass index
FVC: Forced vital capacity
GADD45A: Growth arrest and DNA-damage-inducible, alpha
H3K79: Lysine 79 of histone H3
HRQoL: Health related quality of life
HSkMSCs: Human skeletal muscle satellite cells
LCADL: London Chest Activity of Daily Living Scale
mMRC: Modified medical research council dyspnoea score
mRNA: m Ribonucleic acid
PA: Physical activity
PaCO_2_: Arterial carbon dioxide partial pressure
PA_L_: Physical activity assessed with the LCADL
PaO_2_: Arterial oxygen partial pressure
PA_V_: Physical activity assessed with the Voorrips questionnaire
QMVC: Quadriceps maximal voluntary contraction
qPCR: Real time polymerase chain reaction
ROS: Reactive oxidative species
RP: Rank products
SGRQ: Saint George’s respiratory questionnaire
si RNA: Small interferon ribonucleic acid
ΔCT: Delta cycle threshold
