# Supplementary material for: 2D-DIGE proteomic analysis of vastus lateralis from COPD patients with low and normal fat free mass index and healthy controls
Source: Respir Res. 2017 May 3;18:81. doi: 10.1186/s12931-017-0525-x (PMC5415759; doi:10.1186/s12931-017-0525-x)
Supplement: Additional file 1: — A detailed methodology is provided: Table S1: Differentially expressed spots between different groups comparing COPDN vs C, COPDL vs C and COPDL vs COPDN using the twodimensional fluorescence difference in gel electrophoresis (2D-DIGE) technology. (DOCX 64 kb) [file 12931_2017_525_MOESM1_ESM.docx]

**Additional file 1**

**2D-DIGE proteomic analysis of *vastus lateralis* from COPD patients with low and normal fat free mass index and healthy controls**

*Ramzi Lakhdar^(1)^, Ellen M Drost^(1)^, William MacNee^(1)^, Ricardo Bastos^(2)†^and Roberto A Rabinovich^(1)*†^*

**Ramzi Lakhdar^(1)^**

Email: [lakhdarramzi@yahoo.fr](mailto:lakhdarramzi@yahoo.fr)

**Ellen M Drost^(1)^**

Email: [edrost@ed.ac.uk](mailto:edrost@ed.ac.uk)

**William MacNee^(1)^**

Email: [w.macnee@ed.ac.uk](mailto:w.macnee@ed.ac.uk)

**Ricardo Bastos^(2)†^**

Email: [rbastosbach@gmail.com](mailto:rbastosbach@gmail.com)

**Roberto A Rabinovich^(1)†*^**

^*^Corresponding author

Email: [roberto.rabinovich@ed.ac.uk](mailto:%20roberto.rabinovich@ed.ac.uk%20)

ELEGI Colt Laboratory, Centre for Inflammation Research. The Queen`s Medical Research Institute, University of Edinburgh. 47 Little France Crescent. EDINBURGH, Scotland U.K. EH16 4TJ.

^(1)^ELEGI Colt Laboratory, Centre for Inflammation Research. The Queen`s Medical Research Institute, University of Edinburgh. Scotland, U.K.

*^(2)^*IDIBAPS, University of Barcelona, Spain.

*^†^*Senior co-authors

**METHODS**

**Study Group**

Subjects were considered ex-smokers if they had not smoked for at least 6 month.

All patients were treated with short- and long-acting bronchodilators and inhaled corticosteroids. No patients were taking oral corticosteroids or drugs with potential effects on the muscle. They were clinically stable at the time of the study, without an exacerbation or oral steroid treatment in the previous six weeks. None of the patients had significant co-morbidities.

**MEASUREMENTS**

**Lung function**

Spirometry was measured (Alpha Spirometer; Vitalograph, Buckingham, UK) according to American Thoracic Society/European Respiratory Society standards in all subjects [[1](#_ENREF_1)] before and after the administration of 2.5 mg of nebulised salbutamol. Arterial blood gases were measured (Ciba Corning 800, USA).

**Body composition**

Body composition was estimated by a leg-to-leg bioelectric impedance device (TBF-300M, TANITA Corporation, Tokyo, Japan) while subjects were in supine position. Fat free mass index was obtained by dividing FFM in Kg by height^2^. Low FFMI was defined as <16 kg.m^-2^ for male and < 15 kg.m^-2^ for female COPD patients [[2](#_ENREF_2)].

**Exercise tolerance and muscle strength**

As a measure of exercise tolerance all 27 participants in the present study performed an encouraged 6MWT according to ATS guidelines[[3](#_ENREF_3)] . As a measure of muscle function [[4](#_ENREF_4)], muscle strength was assessed as the maximal isometric quadriceps voluntary contraction (QMVC) using a strain gauge dynamometer (Chatillon® K-MSC 500, Ametek, Florida). Subjects were asked to sit in a purpose-built chair with an inextensible strap connecting the ankle to a strain gauge; knees were flexed to 90°; the strain gauge and couplings were all aligned to ensure that the contraction was isometric. After a previous muscle warm up QMVC was performed 3 to 4 times with vigorous encouragement and rests between contractions; the biggest effort recorded was used for analysis.

**Health related quality of life (HRQoL) and physical activity level**

HRQoL was assessed using the St. George’s Respiratory Questionnaire [[5](#_ENREF_5)]. Physical activity level was assessed using the Voorrips physical activity questionnaire in the whole population participating in the study [[6](#_ENREF_6)]. Moreover, COPD patients activities of daily living was assessed specifically with the London Chest Activity of Daily Living Scale (LCADL)[[7](#_ENREF_7)].

**Muscle biopsy**

Samples were divided and processed as follows: a) fixed in formaldehyde, embedded in paraffin for immunohistochemistry; b) RNA stabilization reagent (RNAlater®, Ambion, Inc., USA) and stored at -20^o^C for RNAextraction; c) immediately frozen in liquid nitrogen and stored at -80^o^Cfor protein extraction.

**Fibre type typification**

Sections were heated to 125^o^C for 30 s and allowed to cool to 90^o^C for 10 s. Sections were washed in running tap water then placed on a Leica Vision Biosystems Bond max immmunostaining robot and stained as follows. Sections were incubated with 3% H_2_O_2_ for 10 min washed in water then TBS/Tween 0.1%. Following incubation with 20% normal goat serum in TBS/Tween the sections were incubated for 120 min with mouse anti myosin Type I at 1:2000 dilution (Dako), following further washes slides were incubated with goat anti Mouse fab peroxidase at 1:500 in NGS/Tween for 30 min. Following washing slides were incubated with Tyramide Cy5 (Blue) (Perkin Elmer) for 10 min and washed. Using antibody elution using HIER as described by Toth et al [[8](#_ENREF_8)] sections for Type II co-localisation were retrieved for 10 min in Bond ER2 epitope retrieval solution followed by 15 min in 0.5mg/ml trypsin (Sigma) in Tris/CaCl2 buffer at 37 C. Sections were incubated with 3% H_2_O_2_ for 10 min washed in water then TBS/Tween. Following incubation with 20% Normal goat serum in TBS/Tween the sections were incubated for 120 min with mouse anti myosin Type II (1:5000) following further washes slides were incubated with goat anti Mouse fab peroxidase at 1:500 in NGS/Tween for 30 mins. Following washing, slides were incubated with Tyramide Cy3 (Red) (Perkin Elmer) for 10 min and counterstained with DAPI. Tiled images of the entire section were acquired using a Zeiss 710 confocal microscope. Five images per patient were included in the analysis. A total of 959.6±146.4; 715.0±89.2 and 918.6±95.2 fibres in Control subjects, COPD_N_ and COPD_L_ respectively (p=ns) were assessed. Type I, Type II and hybrid (identified by the two antibodies) fibres were counted using a manual tag protocol using Media Cybernetics Image pro Plus (Image-Pro Plus, Media Cybernetics, Inc. Bethesda, MD. USA) and expressed as a proportion of total fibres assessed.

***Vastus lateralis* muscle protein extraction**

*Vastus lateralis* muscle (~0.1 g) from each sample was cut into small pieces with a scalpel and transferred in metal bead tubes. 50µl of extracting buffer (0.3% Sodium carbonate, 0.5% Sodium hydrogen carbonate and 0.6% CHAPS, containing phosphatase inhibitors, protease inhibitors and benzonase) was added for 15 mg of each tissue sample. Samples were vortexed on ice, transferred into a centrifuge tube and centrifuged at 7000g during 30 sec. Supernatants were placed into a new tubes and the protein concentration determined by the Bradford method (Bio-Rad Laboratories, Hercules, CA) and subsequently precipitated with methanol.

**Two-dimensional difference gel electrophoresis (2D-DIGE)**

***Preparation of samples and CyDye labeling***

Protein sample buffer was exchanged into 2-D cell lysis buffer (30 mMTris-HCl, pH 8.8, containing 7 M urea, 2 Mthiourea and 4% CHAPS). Protein concentration was measured using Bio-Rad protein assay method (Hercules, CA).Protein labeling with cyanine dyes (Cy2, Cy3 and Cy5) was performed according to the manufacturer’s instructions (GE Healthcare). For each sample, 30 μg of protein was mixed with 1.0 μl of diluted CyDye, and kept in the dark on ice for 30 min. The labeling reaction was stopped by adding 1.0 μl of 10 mM Lysine to each sample, and incubating in the dark on ice for an additional 15 min.

***IEF and SDS-PAGE***

An equal amount of paired samples of each group labeled with Cy3 and Cy5 were dissolved in sample buffer (7 M urea, 2 M thiourea, 4% CHAPS, 20 mg/ml DTT, 1% Pharmalytes). An equal amount of all samples included in the study was labeled with Cy2 and used as an internal standard. The labeled samples were mixed together and subjected to isoelectric focusing (IEF, pH 3-10) in the first dimension and to SDS-PAGE in the second dimension following the protocols provided by the manufacturer. Several gels were run comparing samples from two subjects belonging to two different groups. Working with the 3 groups (COPD_L_, COPD_N_, and C), each gel runs two samples from two patients belonging to different groups out of the three (COPD_L_, COPD_N_, C). In our experimental design, part of the samples of a group was labelled with Cy3 and the remaining with Cy5 to dilute the labelling effect.

***Image Scan and Data Analysis***

Gels were scanned immediately after SDS-PAGE using Typhoon TRIO (GE Healthcare). Images were analyzed by Image QuantTL software and subjected to in-gel analysis and cross-gel analysis using DeCyder 6.5 software (GE Healthcare). The fold change of the protein expression levels was obtained from in-gel DeCyder analysis.

***Spot Picking and Trypsin Digestion***

Spots of interest were picked by Ettan Spot Picker (GE Healthcare) based on the in-gel analysis and spot-picking design by DeCyder software.

Proteins in the gel spots were digested with modified porcine trypsin protease (Trypsin Gold, Promega). The digested tryptic peptides were desalted by Zip-tip C18 (Millipore). Peptides were eluted from the Zip-tip with 0.5μl of matrix solution (α-cyano-4-hydroxycinnamic acid, 5 mg/ml in 50% acetonitrile, 0.1% trifluoroacetic acid, 25 mM ammonium bicarbonate) and spotted on the AB SCIEX MALDI plate (Opti-TOF 384 Well Insert, AB SCIEX, Framingham, MA).

***Mass Spectrometry***

MALDI-TOF (matrix-assisted laser desorption ionization time of flight) and TOF/TOF (tandem MS/MS) were performed on an AB SCIEX TOF/TOF 5800 System (AB SCIEX, Framingham, MA). MALDI-TOF mass spectra were acquired in reflectron positive ion mode, averaging 4000 laser shots per spectrum. TOF/TOF tandem MS fragmentation spectra were acquired for each sample, averaging 4000 laser shots per fragmentation spectrum on each of the 10 most abundant ions present in each sample (excluding trypsin autolytic peptides and other known background ions).

***Database Search***

Both the resulting peptide mass and the associated fragmentation spectra were submitted to GPS Explorer workstation equipped with MASCOT search engine (Matrix Science, Boston, CA) to interrogate the Swiss-Prot database. Searches were performed without constraining protein molecular weight or isoelectric point, with variable carbamidomethylation of cysteine and oxidation of methionine residues, and with one missed cleavage also allowed in the search parameters. Candidates with either protein score C.I% or Ion C.I% greater than 95 were considered significant.

**Cell culture**

Medium was supplemented with the provided foetal bovine Serum (5%), Skeletal Muscle Cell Growth Supplement (SKMCGS) (1%) and Penicillin/Streptomycin solution (P/S solution) (1%) vials and cells were incubated at 37°C with 5% CO2. Myoblasts were differentiated into myotubes as previously described [[9](#_ENREF_9)] by switching the cells into serum-low adding 2% horse serum instead of the foetal bovine serum to the same medium. Prior to switching to serum-low medium, the cultures were rinsed with Dulbecco's Phosphate-Buffered Saline (DPBS 1x –GIBCO, life technologies).

**DOT1L gene kockdown in HSkMSC by siRNA**

***siRNA design***

Knockdown of DOT1L expression was confirmed using quantitative reverse transcription-PCR and westernblot as below detailed. Non-targeting control siRNA (Ambion® Silencer® Negative Control #1 siRNA L/N: 1212008) which has no significant sequence similarity to human gene sequenceswas purchased (Ambicon, Life Technologies).

The following was used as siRNA DOT1L targeting sequence (5’->3’) as it is from the data sheet provided with the reagents:

Sense GCUCGCUAUGGAGAAUUACtt; Antisense GUAAUUCUCCAUAGCGAGCtt

***siRNA cell transfection***

6uL of Lipofectamine2000 Reagent and 3uL siRNA (10uM) were diluted separately in 150uL serum free antibiotic free (SFAF) medium in a FACS (Fluorescence activated cell sorted) tubes and left at room temperature for 15 minutes before combining 150 uL from each solution which gives final concentration of 30 pmol siRNA per well in a six well plate. This mixture was left to complex for 30 minutes under gentle agitation. Meanwhile, cells were washed two times with DPBS and 0.7ml SFAF medium was added to cells. The complex (Lipofectamine2000 Reagent/siRNA) was added wise to cells and gently agitated to mix then the cells were put back in the incubator. After 7 hours the medium was replaced with complete medium (serum and antibiotics) and the cells were incubated to grow for 48 hours. The growth medium was then discarded and the cells were washed twice with DPBS before adding the differentiation medium to differentiate the cells for 24 hours. The cells were harvested adding 300 ul trypsin per well, trypsin activity was ended by the added medium with FCS. After centrifugation the cells were stored at -80°C to proceed later to RNA and protein extraction. Control cultures were similarly prepared, but without addition of siRNA (control), or transfected using the non-targeting control siRNA (negative control).

**Quantitative RT-PCR**

RNA, 18S Ribosomal 5 (18S) was used for normalization of cDNA input. The thermal cycling conditions were as follows: initial denaturation at 95°C for 15 min, 45 cycles of 95°C for 15 s, 53°C for 15 s and 72°C for 30 s, followed by dissociation step analysis (72–99°C with 1°C increments). The amplification was specific as judged by dissociation temperature analysis. The experiments were performed in duplicate and three times. The following oligonucleotides were used as primers:

DOT1L_For AAACTCAGGGAGGAACAGGAGG

DOT1L_Rev TGAGGGGATCTGTAGGCATCCTG

CDKN1A_For TGTGTGCTGCGTTCACAGGTG

CDKN1A_Rev ATCTGTCATGCTGGTCTGCCG

**Western Blot Analysis**

15 μg of protein was resolved by sodium-dodecyl sulfate-polyacrylamide gel electrophoresis on NuPAGE® Novex® 4-12% Bis-Tris Gels (NP0335BOX,Life Technologies Corporation). Proteins were transferred to Immobilon-P PVDF membranes (Millipore, Billerica, MA), blocked with 5% dry milk (Bio-Rad, München, Germany) in TBS (Sigma) during 1 hour at room temperature and then probed with primary antibodies against DOT1L (Novus Biologicals, NB100-40845) and p21 (ab79601) (Abcam, Bristol, UK) overnight at 4^o^C. Membranes were washed with TBS and incubated with the appropriate secondary antibody during 1 hour at room temperature. Proteins were then visualized using the ECL Detection System (Pierce, Rockford, IL) as per the manufacturer’s instructions.

**REFERENCES**

1. Miller MR, Hankinson J, Brusasco V, Burgos F, Casaburi R, Coates A, Crapo R, Enright P, van der Grinten CP, Gustafsson P, et al: **Standardisation of spirometry.***Eur Respir J* 2005, **26:**319-338.

2. Schols AM, Broekhuizen R, Weling-Scheepers CA, Wouters EF: **Body composition and mortality in chronic obstructive pulmonary disease.***AmJClinNutr* 2005, **82:**53-59.

3. Holland AE, Spruit MA, Troosters T, Puhan MA, Pepin V, Saey D, McCormack MC, Carlin BW, Sciurba FC, Pitta F, et al: **An official European Respiratory Society/American Thoracic Society technical standard: field walking tests in chronic respiratory disease.***Eur Respir J* 2014, **44:**1428-1446.

4. Spruit MA, Gosselink R, Troosters T, Kasran A, Gayan-Ramirez G, Bogaerts P, Bouillon R, Decramer M: **Muscle force during an acute exacerbation in hospitalised patients with COPD and its relationship with CXCL8 and IGF-I.***Thorax* 2003, **58:**752-756.

5. Jones PW, Quirk FH, Baveystock CM, Littlejohns P: **A Self-complete Measure of Health Status for Chronic Airflow Limitation.***American Review of Respiratory Disease* 1992, **145:**1321-1327.

6. Voorrips LE, Ravelli AC, Dongelmans PC, Deurenberg P, Van Staveren WA: **A physical activity questionnaire for the elderly.***MedSciSports Exerc* 1991, **23:**974-979.

7. Garrod R, Bestall JC, Paul EA, Wedzicha JA, Jones PW: **Development and validation of a standardized measure of activity of daily living in patients with severe COPD: the London Chest Activity of Daily Living scale (LCADL).***Respir Med* 2000, **94:**589-596.

8. Toth ZE, Mezey E: **Simultaneous visualization of multiple antigens with tyramide signal amplification using antibodies from the same species.***J Histochem Cytochem* 2007, **55:**545-554.

9. Danoviz ME, Yablonka-Reuveni Z: **Skeletal muscle satellite cells: background and methods for isolation and analysis in a primary culture system.***Methods Mol Biol* 2012, **798:**21-52.

**Table S1: Differentially expressed spots between different groups comparing COPD_N_ vs C, COPD_L_ vs C and COPD_L_ vs COPD_N_ using the two-dimensional fluorescence difference in gel electrophoresis (2D-DIGE) technology.**

|  |  |  | **COPD_N_ vs C** | |  | **COPD_L_ vs C** | |  | **COPD_L_ vs COPD_N_** | |
| --- | --- | --- | --- | --- | --- | --- | --- | --- | --- | --- |
| **Assigned #** | **Original No.** |  | **p-value** | **Av. Ratio** |  | **p-value** | **Av. Ratio** |  | **p-value** | **Av. Ratio** |
| **1** | 22 |  | 0.16 | 1.54 |  | 0.014 | -1.45 |  | 0.0065 | -2.25 |
| **2** | 146 |  | 0.16 | 1.42 |  | 0.95 | -1.02 |  | 0.14 | -1.44 |
| **3** | 46 |  | 0.8 | -1.06 |  | 0.74 | -1.07 |  | 0.97 | -1.01 |
| **4** | 123 |  | 0.67 | -1.05 |  | 0.36 | 1.1 |  | 0.22 | 1.15 |
| **5** | 125 |  | 0.51 | -1.15 |  | 0.75 | -1.1 |  | 0.62 | 1.05 |
| **6** | 111 |  | 0.55 | -1.14 |  | 0.19 | -1.25 |  | 0.21 | -1.09 |
| **7** | 223 |  | 0.049 | 1.39 |  | 0.055 | 1.33 |  | 0.8 | -1.05 |
| **8** | 194 |  | 0.054 | 1.45 |  | 0.53 | 1.05 |  | 0.08 | -1.38 |
| **9** | 267 |  | 0.00012 | 1.31 |  | 3.50E-07 | 1.38 |  | 2.60E-01 | 1.05 |
| **10** | 290 |  | 0.06 | 1.53 |  | 0.66 | -1.07 |  | 0.028 | -1.64 |
| **11** | 222 |  | 0.033 | 1.41 |  | 0.029 | 1.39 |  | 0.92 | -1.02 |
| **12** | 353 |  | 0.00039 | 1.36 |  | 0.00069 | 1.28 |  | 0.39 | -1.06 |
| **13** | 323 |  | 0.044 | 1.68 |  | 0.14 | 1.22 |  | 0.23 | -1.38 |
| **14** | 327 |  | 0.025 | 1.71 |  | 0.066 | 1.26 |  | 0.22 | -1.36 |
| **15** | 328 |  | 0.0095 | 1.75 |  | 0.043 | 1.29 |  | 0.16 | -1.35 |
| **16** | 329 |  | 0.0002 | 2.03 |  | 0.001 | 1.6 |  | 0.21 | -1.26 |
| **17** | 417 |  | 0.17 | -1.23 |  | 0.022 | 1.36 |  | 0.0053 | 1.67 |
| **18** | 424 |  | 0.38 | -1.08 |  | 0.0035 | 1.43 |  | 0.0078 | 1.54 |
| **19** | 425 |  | 0.61 | -1.02 |  | 0.015 | 1.34 |  | 0.038 | 1.37 |
| **20** | 410 |  | 0.58 | 1.21 |  | 0.0054 | 1.37 |  | 0.29 | 1.13 |
| **21** | 504 |  | 0.044 | -1.33 |  | 0.32 | -1.12 |  | 0.15 | 1.2 |
| **22** | 548 |  | 0.0049 | -1.4 |  | 0.033 | -1.25 |  | 0.32 | 1.12 |
| **23** | 554 |  | 0.00091 | -1.63 |  | 0.058 | -1.28 |  | 0.072 | 1.27 |
| **24** | 651 |  | 0.00023 | 2.1 |  | 0.02 | 1.26 |  | 0.0021 | -1.67 |
| **25** | 654 |  | 0.028 | 1.69 |  | 0.069 | 1.26 |  | 0.19 | -1.34 |
| **26** | 521 |  | 0.015 | 1.35 |  | 7.80E-05 | 1.71 |  | 3.60E-03 | 1.27 |
| **27** | 524 |  | 0.013 | 1.29 |  | 2.00E-05 | 1.61 |  | 8.10E-03 | 1.25 |
| **28** | 525 |  | 0.0033 | 1.87 |  | 0.06 | 1.3 |  | 0.053 | -1.44 |
| **29** | 663 |  | 0.0023 | 2.09 |  | 0.098 | 1.24 |  | 0.0085 | -1.68 |
| **30** | 666 |  | 0.0008 | 2.29 |  | 0.16 | 1.19 |  | 0.003 | -1.92 |
| **31** | 624 |  | 0.096 | 1.78 |  | 0.87 | -1.05 |  | 0.078 | -1.86 |
| **32** | 627 |  | 0.22 | 1.86 |  | 0.52 | -1.18 |  | 0.11 | -2.18 |
| **33** | 635 |  | 9.80E-05 | 1.57 |  | 0.012 | 1.23 |  | 0.013 | -1.27 |
| **34** | 723 |  | 0.012 | -1.41 |  | 0.77 | 1.03 |  | 0.0031 | 1.45 |
| **35** | 784 |  | 0.0074 | -1.48 |  | 0.17 | -1.2 |  | 0.028 | 1.23 |
| **36** | 851 |  | 0.07 | -1.86 |  | 0.86 | -1.02 |  | 0.013 | 1.83 |
| **37** | 777 |  | 0.053 | 1.5 |  | 0.96 | -1.07 |  | 0.011 | -1.61 |
| **38** | 791 |  | 0.038 | 1.64 |  | 0.98 | -1.08 |  | 0.0081 | -1.77 |
| **39** | 771 |  | 0.021 | 1.94 |  | 0.057 | 1.4 |  | 0.28 | -1.38 |
| **40** | 756 |  | 0.043 | 1.63 |  | 0.041 | 1.61 |  | 0.94 | -1.02 |
| **41** | 757 |  | 0.0023 | -1.67 |  | 0.00043 | -1.59 |  | 0.41 | 1.05 |
| **42** | 762 |  | 0.00073 | -1.68 |  | 0.00023 | -1.58 |  | 0.37 | 1.06 |
| **43** | 775 |  | 0.002 | -1.58 |  | 0.00013 | -1.52 |  | 0.47 | 1.04 |
| **44** | 785 |  | 0.002 | -1.49 |  | 0.0001 | -1.48 |  | 0.68 | 1.01 |
| **45** | 792 |  | 0.0053 | -1.4 |  | 7.50E-05 | -1.5 |  | 5.40E-01 | -1.07 |
| **46** | 799 |  | 0.0022 | -1.55 |  | 4.00E-05 | -1.65 |  | 7.20E-01 | -1.06 |
| **47** | 940 |  | 0.68 | 1.13 |  | 0.042 | -1.56 |  | 0.022 | -1.76 |
| **48** | 941 |  | 0.74 | 1.18 |  | 0.023 | -2.21 |  | 0.025 | -2.6 |
| **49** | 942 |  | 0.52 | 1.36 |  | 0.029 | -2.17 |  | 0.018 | -2.95 |
| **50** | 912 |  | 0.052 | 1.64 |  | 0.16 | 1.26 |  | 0.25 | -1.3 |
| **51** | 925 |  | 0.022 | 2.23 |  | 0.12 | 1.22 |  | 0.071 | -1.84 |
| **52** | 927 |  | 0.034 | 2.16 |  | 0.23 | 1.13 |  | 0.072 | -1.91 |
| **53** | 963 |  | 0.13 | 1.65 |  | 0.36 | 1.05 |  | 0.21 | -1.58 |
| **54** | 964 |  | 0.11 | 1.66 |  | 0.58 | -1.06 |  | 0.1 | -1.75 |
| **55** | 965 |  | 0.14 | 1.46 |  | 0.49 | 1.02 |  | 0.19 | -1.43 |
| **56** | 966 |  | 0.14 | 1.62 |  | 0.28 | 1.13 |  | 0.26 | -1.43 |
| **57** | 1035 |  | 0.47 | 1.37 |  | 0.071 | -1.78 |  | 0.023 | -2.43 |
| **58** | 1007 |  | 0.0076 | 2 |  | 0.0064 | 1.47 |  | 0.23 | -1.36 |
| **59** | 1006 |  | 0.017 | 1.81 |  | 0.39 | 1.1 |  | 0.047 | -1.65 |
| **60** | 1041 |  | 0.17 | 1.5 |  | 0.029 | -1.63 |  | 0.00086 | -2.44 |
| **61** | 1088 |  | 0.3 | -1.49 |  | 0.7 | -1.04 |  | 0.11 | 1.43 |
| **62** | 1173 |  | 0.36 | 1.21 |  | 0.97 | -1.01 |  | 0.33 | -1.22 |
| **63** | 1239 |  | 0.29 | -1.16 |  | 0.26 | -1.17 |  | 0.96 | -1.01 |
| **64** | 1145 |  | 0.061 | -1.5 |  | 0.41 | -1.2 |  | 0.15 | 1.24 |
| **65** | 1247 |  | 0.034 | -1.38 |  | 0.12 | -1.21 |  | 0.17 | 1.14 |
| **66** | 1297 |  | 1.50E-05 | 1.81 |  | 3.70E-09 | 1.86 |  | 5.90E-01 | 1.03 |
| **67** | 1304 |  | 0.21 | 1.13 |  | 6.70E-05 | 1.48 |  | 1.10E-02 | 1.3 |
| **68** | 1337 |  | 8.20E-06 | 1.73 |  | 4.50E-07 | 1.71 |  | 9.40E-01 | -1.01 |
| **69** | 1356 |  | 0.0023 | -1.46 |  | 0.16 | -1.14 |  | 0.029 | 1.28 |
| **70** | 1357 |  | 0.00096 | -1.38 |  | 0.43 | -1.08 |  | 0.019 | 1.29 |
| **71** | 1364 |  | 0.66 | -1.08 |  | 0.15 | 1.31 |  | 0.065 | 1.42 |
| **72** | 1324 |  | 0.2 | 1.17 |  | 0.51 | -1.12 |  | 0.03 | -1.31 |
| **73** | 1392 |  | 0.24 | -1.39 |  | 0.27 | 1.27 |  | 0.033 | 1.77 |
| **74** | 1484 |  | 0.074 | -1.23 |  | 0.77 | 1 |  | 0.2 | 1.23 |
| **75** | 1513 |  | 0.46 | 1.11 |  | 0.00026 | 1.53 |  | 0.017 | 1.38 |
| **76** | 1472 |  | 0.0045 | 1.39 |  | 0.00025 | 1.54 |  | 0.084 | 1.1 |
| **77** | 1461 |  | 0.01 | 1.54 |  | 0.0013 | 1.5 |  | 0.98 | -1.03 |
| **78** | 1446 |  | 0.019 | 1.89 |  | 0.016 | 1.43 |  | 0.31 | -1.32 |
| **79** | 1615 |  | 0.68 | 1.33 |  | 0.099 | -1.43 |  | 0.13 | -1.9 |
| **80** | 1608 |  | 0.95 | 1.21 |  | 0.039 | -1.77 |  | 0.11 | -2.14 |
| **81** | 1758 |  | 0.079 | 1.74 |  | 0.45 | 1.1 |  | 0.14 | -1.59 |
| **82** | 1742 |  | 0.58 | 1.32 |  | 0.71 | 1.05 |  | 0.68 | -1.25 |
| **83** | 1713 |  | 0.067 | 1.76 |  | 0.19 | 1.17 |  | 0.2 | -1.51 |
| **84** | 1751 |  | 0.55 | 1.23 |  | 0.18 | -1.53 |  | 0.076 | -1.89 |
| **85** | 1874 |  | 0.0014 | -2.85 |  | 0.0043 | -2.12 |  | 0.15 | 1.35 |
| **86** | 1903 |  | 0.0017 | -3.05 |  | 0.0024 | -2.47 |  | 0.2 | 1.24 |
| **87** | 1911 |  | 0.0018 | -2.38 |  | 0.0009 | -2.05 |  | 0.24 | 1.16 |
| **88** | 1916 |  | 0.0038 | -2.64 |  | 0.0014 | -2.63 |  | 0.44 | 1.01 |
| **89** | 1878 |  | 0.014 | -2.64 |  | 0.2 | -1.69 |  | 0.036 | 1.56 |
| **90** | 1892 |  | 0.0057 | -2.64 |  | 0.11 | -1.47 |  | 0.13 | 1.8 |
| **91** | 2088 |  | 0.61 | -1.11 |  | 0.0048 | -2.38 |  | 0.049 | -2.13 |
| **92** | 2104 |  | 0.015 | -1.4 |  | 0.00035 | -1.58 |  | 0.35 | -1.13 |
| **93** | 611 |  | 0.11 | 1.67 |  | 0.66 | -1.15 |  | 0.028 | -1.93 |
| **94** | 632 |  | 0.0037 | 1.69 |  | 0.12 | 1.15 |  | 0.0046 | -1.47 |
| **95** | 1123 |  | 0.76 | -1.27 |  | 0.54 | 1 |  | 0.31 | 1.27 |
| **96** | 1211 |  | 0.86 | -1.17 |  | 0.52 | 1 |  | 0.34 | 1.17 |
|  | **Total** |  | **50** |  |  | **41** |  |  | **37** |  |

List with 96 protein spots selected using the DeCyder software. 50 spots were differentially expressed comparing COPD_N_ vs C, 41 spots were differentially expressed comparing COPD_L_ vs Control COPD and 37 spots were differentially expressed comparing COPD_L_ vs COPD_N_.Student t test was used to compare between different groups, p-value <0.05 significant (highlighted with pattern in the table). Av. Ratio: Average. Ratio; fold change a positive value means increased ratio, a negative value means decreased ratio. Original No: spots number that DeCyder software displays in the overall analysis of the gels. Assigned #: new number assigned to spots selected in the comparative analysis.
